# Prevalence, determinants and outcomes of traditional, complementary and alternative medicine use for hypertension among low-income households in Malaysia and the Philippines

**DOI:** 10.1186/s12906-022-03730-x

**Published:** 2022-09-30

**Authors:** Lia M. Palileo-Villanueva, Benjamin Palafox, Arianna Maever L. Amit, Veincent Christian F. Pepito, Fadhlina Ab-Majid, Farnaza Ariffin, Dina Balabanova, Mohamad-Rodi Isa, Nafiza Mat-Nasir, Mazapuspavina My, Alicia Renedo, Maureen L. Seguin, Khalid Yusoff, Antonio L. Dans, Martin Mckee

**Affiliations:** 1 College of Medicine, University of the Philippines Manila, Manila, Philippines; 2 Faculty of Public Health and Policy, London School of Hygiene and Tropical Medicine, Centre for Global Chronic Conditions, 15-17 Tavistock Place, London, WC1H 9SH UK; 3 School of Medicine and Public Health, Ateneo de Manila University, Pasig City, Philippines; 4 Faculty of Medicine, Universiti Teknologi MARA, Sungai Buloh, Malaysia; 5 Faculty of Medicine and Health Sciences, UCSI University, Kuala Lumpur, Malaysia

**Keywords:** Hypertension, Blood pressure, Traditional, complementary and alternative medicine, Non-communicable disease control, Malaysia, Philippines

## Abstract

**Background:**

Traditional, complementary and alternative medicine (TCAM) is used to treat a broad range of conditions. In low- and middle-income countries (LMICs), TCAM use is particularly common among those with low socio-economic status. To better understand the patterns and impact of TCAM use on the management of non-communicable diseases in these populations, this study examines the prevalence and characteristics of TCAM use for hypertension, its determinants, and its association with hypertension management outcomes and wellbeing among low-income adults in two Southeast Asian countries at different levels of economic and health system development, Malaysia and the Philippines.

**Methods:**

We analysed cross-sectional data from 946 randomly selected adults diagnosed with hypertension from low-income rural and urban communities in Malaysia (*n* = 495) and the Philippines (*n* = 451). We compared the prevalence, characteristics and household expenditure on TCAM use between countries and used multi-level, mixed-effects regression to estimate associations between TCAM use and its determinants, and five hypertension management outcomes and wellbeing.

**Results:**

The prevalence of TCAM use to manage hypertension was higher in the Philippines than in Malaysia (18.8% vs 8.8%, *p* < 0.001). Biologically-based modalities, e.g. herbal remedies, were the most common type of TCAM used in both countries, mainly as a complement, rather than an alternative to conventional treatment. Households allocated around 10% of health spending to TCAM in both countries. Belief that TCAM is effective for hypertension was a positive predictor of TCAM use, while belief in conventional medicine was a negative predictor. TCAM use was not strongly associated with current use of medications for hypertension, self-reported medication adherence, blood pressure level and control, or wellbeing in either country.

**Conclusions:**

A small, but significant, proportion of individuals living in low-income communities in Malaysia and the Philippines use TCAM to manage their hypertension, despite a general lack of evidence on efficacy and safety of commonly used TCAM modalities. Recognising that their patients may be using TCAM to manage hypertension will enable health care providers to deliver safer, more patient-centred care.

**Supplementary Information:**

The online version contains supplementary material available at 10.1186/s12906-022-03730-x.

## Background

Every day large numbers of people use traditional, complementary and alternative medicines (TCAM) to support their health and wellbeing [1–3]. The World Health Organization (WHO) defines *traditional medicine* as the “sum total of the knowledge, skill and practices based on the theories, beliefs and experiences indigenous to different cultures, used in the maintenance of health as well as in the prevention, diagnosis, improvement or treatment” of illness; while *complementary* or *alternative* medicine encompasses a broad set of health care practices that are not considered within the conventional Western allopathic medical model [4]. The extent to which these services and products are used is considerable, but varies widely from country to country. A systematic review covering mainly high- and upper-middle income countries found that 26% of the general population in the United Kingdom had used any form of TCAM in the previous year, but this rose to 76% in Japan [1]. Except in Australia, TCAM use was higher in non-Western countries, [1] reflecting differences in the cultural embeddedness of TCAM from context to context [5].

The dominant forms of TCAM used also varies, particularly across low- and middle-income countries (LMICs), as shown by two systematic reviews conducted across Sub-Saharan Africa and ASEAN member states [2, 3]. These found that herbs and supplements were the common modalities used in both regions [2, 3]. Manipulative and body-based therapies, such as massage, were also common; as were mind–body methods, such as prayer and meditation; while the frequent use of alternative medical systems, including traditional Chinese medicine, and energy therapies like acupuncture, was noted only in Southeast Asia [2, 3].

Both reviews also illustrate the broad range of health concerns for which these treatments were used. In Sub-Saharan Africa, TCAM was used for an especially wide array of conditions, including reproductive and sexual health concerns, communicable diseases, ophthalmic conditions, musculoskeletal pain and to mend broken bones [2]. These are in addition to the applications found to be common across both regions, including for HIV/AIDS, mental health, neurological conditions, and a range of other non-communicable diseases (NCDs), including cancer, diabetes, asthma and hypertension [2, 3].

The relationship between TCAM and conventional medicine also varies. An average of 55% of general populations across Sub-Saharan Africa use TCAM alongside conventional medicine [2]. In Southeast Asia, detailed national findings from Malaysia found that 41% of users do so concurrently with conventional treatment, while 40% choose to seek treatment initially from TCAM providers before obtaining conventional care, while the remainder use TCAM only as an alternative to conventional treatment [6].

By 2018, 150 of 170 World Health Organization (WHO) member states had formally recognised the use of TCAM in laws, regulations, or institutional structures; however, the degree and means of ensuring the safety and effectiveness of TCAM products and services, and of their integration with conventional medical care differs from country to country [4]. Initiatives, such as the ‘Roadmap for an Association of Southeast Asian Nations (ASEAN) Community (2009–2015)’, have sought to harmonise the production, regulation, marketing and integration of TCAM in the region, [7] again reflecting the cultural significance that non-Western systems of medicine play in these countries [5].

Alongside cultural factors, TCAM use in LMICs is also driven by access. In many of these settings, national health system weaknesses mean that TCAM services are more physically accessible and affordable than conventional healthcare options [8, 9]. Consequently, those suffering from chronic and/or multiple conditions, from poorer households and more remote communities are all more likely to use TCAM products and services [2, 3, 10–12]. These individuals are also some of the most likely to experience the devastating economic and social consequences of ill health [13, 14]. Therefore, understanding the patterns and impacts of TCAM use for poor, chronically ill people in different LMIC contexts is important as these countries work toward reducing health and social inequalities in the push to achieve universal health coverage and the Sustainable Development Goals. To date, few such targeted data exist.

This paper explores the use of TCAM for the management of hypertension by people living in low-income communities in two Southeast Asian countries with different health systems and at different levels of economic development: the Philippines, a lower-middle income nation with a pluralistic health system where a recent study reported that 43% of adults aged 20 to 50 years had used it in the past 6 months; [15] and Malaysia, a richer country with a more developed public healthcare system where 53%-56% of the population reported using TCAM in the previous year [12, 16, 17]. Although the health systems in both countries are composed of a tax-funded public sector and a large private sector funded primarily via user fees at the point of service, these countries were selected in part to study the effect of key health system differences on the management of hypertension in our study population of low-income adults.

The Philippines is a large archipelago of more than 7000 islands, with a population greater than 100 million [18]. While the nation’s economy is one of the fastest growing in the region, nearly a fifth of the population remains impoverished [19]. Public sector health services are delivered by various levels of government, and primary care is the responsibility of municipal and city governments implemented through their networks of rural health units, health centres and barangay (village) health stations. However, access to both public and private care remains inequitable due to the maldistribution of health facilities and staff [18].

In contrast, Malaysia is a multi-cultural and highly urbanised nation with less than a third of the Philippine population [20]. Malaysia’s health system has been noted for its highly developed public sector, and particularly its extensive network of health centres and community clinics that provide good levels of access to free primary care throughout the country [20]. As a result, government services have increasingly served rural populations and the poor, while private services tend to be used more by better-off people who live in urban areas – a pattern of health service use also observed in the Philippines [18, 20].

The difference in national wealth and economic development between the two countries is also reflected in spending on health, with total expenditure in Malaysia being three times the level in the Philippines (i.e. per capita total health expenditure in 2019 was 142 US Dollars in the Philippines vs. 437 US Dollars in Malaysia). While over half of the spending on health in Malaysia is covered by government transfers raised by taxation (51.5%) with another 34.6% paid by out-of-pocket spending from individuals and households, the situation in Philippines is reversed, with 48.6% of health spending coming out-of-pocket and 34.0% covered by government transfers [21].

In the Philippines, a small but significant portion of health care is also financed via social health insurance administered by the Philippine Health Insurance Corporation (PhilHealth), which covers more than 90% of the population, including the poor whose insurance premiums are subsidised by the government. However, the financial protection provided by PhilHealth is limited, as coverage focuses on inpatient care and only the outpatient care poor PhilHealth members, resulting in high levels of out-of-pocket spending [18].

Hypertension, or high blood pressure, is a common chronic condition for which existing conventional medicine offers safe and effective treatments – yet, only a third of affected individuals are able to successfully manage the condition [22]. Hypertension is the leading risk factor for cardiovascular disease (CVD), which in turn, is the key driver of the global burden from NCDs, [23] and particularly in Southeast Asia [24]. In Malaysia, NCDs now account 74% of all disability-adjusted life years lost, and 65% in the Philippines [23]. While several studies have explored the use of TCAM by individuals with hypertension in a number of Southeast Asian countries, [11, 25–30] similar evidence from the Philippines and Malaysia is unavailable or of limited scope. As such, this study primarily aims to describe and compare the use of TCAM specifically for the management of hypertension among low-income individuals. It also seeks to assess the potential determinants of TCAM use for hypertension and the potential effects of TCAM use on hypertension management outcomes. It is hoped that the resulting evidence could inform more equitable and inclusive strategies to improve hypertension management outcomes in both countries, and LMICs more broadly.

## Methods

### Data collection

Data were collected within the Responsive and Equitable Health Systems-Partnership on Non-Communicable Diseases (RESPOND) project, a mixed-methods, longitudinal, observational study on treatment seeking for hypertension in Malaysia and the Philippines, the details of which have been published elsewhere [31].

Briefly, low-income communities (i.e. ‘mukim’ in Malaysia and ‘barangays’ in the Philippines with high proportions of households qualifying for government subsides) were selected in urban and rural strata with probability proportional to size in purposefully selected states (Malaysia) and provinces/cities (Philippines). In Malaysia, these peninsular states included Selangor, Kelantan, Perak and Johor. In the Philippines, urban communities are in the City of Valenzuela in Metro Manila and 8 urban and 15 rural communities in Quezon province. Within selected low-income communities, households were randomly selected either using lists obtained from local government units, or in the absence of suitable records, using the WHO Expanded Programme on Immunisation sampling approach [32].

Selected households were eligible for participation if they were low-income (i.e. self-reported as qualifying for government subsidies under the BR1M programme in Malaysia [33] and the 4P programme in the Philippines, [34] which are both national cash transfer programmes for poverty alleviation, social assistance and development); and if at least one household member was an adult aged 35–70 who either self-reported a history of hypertension diagnosis or were identified as hypertensive during blood pressure screening following a standardised procedure [35]. Those without a self-reported history of hypertension were categorised as hypertensive if either the average systolic or diastolic blood pressure was equal to or above 140 mmHg or 90 mmHg, respectively. One among all hypertensive members in eligible households was randomly selected to participate, resulting in a representative sample from included communities. A priori sample size determination took account of many possible analyses. For example, the ability to detect an cross-country difference in TCAM use, which recent estimates suggest could as large as 13 percentage points (43% in the Philippines, 56% in Malaysia), [3, 15] would require a sample of 461 individuals with hypertension across 21 communities from each country (α = 0.05, power = 0.8 (two-tailed), intra-class coefficient = 0.05, cluster size = 20).

On enrolment, field personnel trained using a demonstration-return demonstration approach administered a questionnaire comprised of validated instruments to participants within their homes to collect information on housing characteristics, socio-economic characteristics of households and participants, and hypertension-related care experiences, practices, knowledge and attitudes [31]. Questions on TCAM use specifically for hypertension were adapted from the international CAM questionnaire (I-CAM-Q), which asked about the types of providers visited and treatments received, the use of herbs and supplements, and self-care practices [36]. Following piloting and due to feasibility, all respondents in the Philippines and a random sub-sample of respondents in Malaysia also provided information on household income and expenditure, and expenditure related to general health and hypertension management. This analysis of TCAM use for hypertension uses cross-sectional baseline data collected in 2018; as such, only participants who self-reported as having been diagnosed with hypertension by a health professional at baseline were included.

### Variables of interest

To assess the determinants of TCAM use among our study population, we evaluate one binary outcome, current use of TCAM, which is defined by self-reports of using any type of TCAM for hypertension, where ‘current’ describes regular use in the preceding 2 weeks. Acknowledging that no general standard exists on what constitutes a TCAM therapy, and the inherent challenges of defining and categorising certain therapies, [37] in our analyses, TCAM services, practices and products, whether used in self-care or rendered by providers, are categorised into the five domains adopted by Wieland et al. in 2011, [38] and as originally developed by the National Center for Complementary and Alternative Medicine in the United States (now known as the National Center for Complementary and Integrative Health): 1) mind–body therapies (e.g. yoga, Tai Chi, meditation), 2) natural and biological-based therapies (e.g. herbs, supplements), 3) manipulative and body-based systems (e.g. massage), 4) energy therapies (e.g. acupuncture, Reiki), and 5) whole medical systems that cut across the previous domains and comprise theories and practices outside of the conventional Western allopathic model (e.g. homeopathy, naturopathy, traditional Chinese medicine) [38]. To assess the potential effect of TCAM use at different stages of the hypertension management pathway, we report and estimate its association with the following internationally standardised indicators: 1) current use of conventional hypertension medication and 2) blood pressure control among those with a self-reported history of hypertension; 3) current adherence among those currently using hypertension medication; 4) average systolic and 5) average diastolic blood pressure measured in mmHg; and, 6) self-reported general wellbeing, rated on a scale ranging from 1 (least satisfied) to 10 (most satisfied). In line with globally accepted survey practice, current use of medication is defined by self-reports of using any type of conventional antihypertensive medication in the past 2 weeks, and control as participants with a history of hypertension whose average systolic and diastolic blood pressure was measured to be less than 140/90 mmHg [39]. Appendix 1 in the Supplemental information provides full definitions of all variables.

### Independent variables and covariates

The selection of variables examined as potential determinants of TCAM use and as covariates for the associations between TCAM use and the six hypertension management outcomes described above was theoretically informed by the CAM Healthcare Model, which was adapted from Andersen’s Behavioural Model of Health Service Use [40]. Following this model, selected variables address the domains of participant demographics (sex, age, marital status), social factors (education, hypertension knowledge), beliefs and values (confidence in health system, receives regular health care), risk perception (belief that TCAM and/or convention medicine is effective to treat hypertension), personal factors (belief that one can do something to maintain health), resources (household wealth, employment), geographic location (urban vs. rural location), and evaluated need for care (self-reported history of NCDs, time since hypertension diagnosis). Appendix 1 in the Supplemental information provides full definitions of all variables.

### Statistical analysis

We present summary statistics of participant and household characteristics, and indicators of TCAM use and hypertension management as percentages and means by country. Wald tests assess differences in proportions or means across countries. The distribution of TCAM types used for hypertension by country is based on the number of times each was mentioned by participants (i.e. participants may report using more than one type of TCAM simultaneously) over the total number of mentions.

We estimated the association between TCAM use and its determinants, and between TCAM use and the six hypertension management outcomes in separate country-specific models for participants with known hypertension history. We used multi-level, mixed-effects logistic (for binary outcomes) and linear (for continuous outcomes) regression models with a community-level random effect to account for clustering arising from unobserved factors that vary by community. According to the CAM healthcare model, such unobserved factors could relate to cultural norms and practices, and the availability and accessibility of TCAM and conventional medical providers, services, products and information [40]. Therefore, our models attempt to control for such important community-level sources of unobserved confounding.

We present both crude and adjusted estimates as odds ratios (for binary outcomes) and regression coefficients (for continuous outcomes) with their 95% confidence intervals. For the multivariable model of TCAM use, each determinant is mutually adjusted for all other potential determinants described above. The models assessing the association between each of the hypertension management outcomes and TCAM use are adjusted for belief in the effectiveness of TCAM and conventional medicine for hypertension, knowledge of hypertension, time since diagnosed with hypertension, history of NCD comorbidity, frequency of health provider visits, sex, age, education, marital/co-habitation status, employment, confidence in health system, belief that one can do something to maintain health, urban–rural location and household wealth. Models assessing the association between TCAM use and hypertension control, systolic and diastolic blood pressure, and wellbeing are further adjusted for current use of antihypertensive medication. We also performed tests of interaction by country using Wald tests for the equality of country-specific adjusted associations (i.e. for each TCAM use determinant, and its effect on each of the hypertension management outcomes) in fully interacted models of the combined country dataset [41].

Summary statistics, crude and adjusted odds ratios and coefficients from regression models, and Wald tests are weighted for sampling probability and adjusted for community-level clustering to account for the sampling design. Therefore, estimates aim to be representative for the low-income populations of the selected states, provinces and cities in the two countries. Appendix 2 in the Supplemental information provides information on the derivation of the probability-based sampling weights.

## Results

Of the 1191 participants categorised as hypertensive and enrolled in the RESPOND study, 946 of these reported having been diagnosed by a health professional for hypertension and were included in this analysis of TCAM use for hypertension, 451 in the Philippines and 495 in Malaysia. We excluded 39 participants (4.1%) from this analysis due to missing data, but they did not appear to differ notably from those included in terms of age, time since diagnosis, marital or employment status; although fewer in the group excluded in the Philippines had any post-secondary education, and in Malaysia the group included more females, were slightly younger and reported fewer NCD comorbidities (see Appendix 3 in the Supplemental information; Appendix 4 provides a completed reporting checklist for cross-sectional studies and flow diagram illustrating the derivation of our analytical sample).

### Sociodemographic and health-seeking characteristics

In both countries, most participants were female, married/cohabitating and not currently employed (Table 1). Less than a third of participants in the Philippines (27.2%) and Malaysia (28.9%) had controlled hypertension, despite most reporting current use of blood-pressure lowering medications (82.4% vs. 88.4%). A higher proportion of participants in Malaysia reported good adherence to their anti-hypertension medications compared to the Philippines (98.8% vs. 65.3%, *p* < 0.001).

**Table 1:** Sociodemographic and health-seeking characteristics of diagnosed hypertensive adults from low-income communities, by country

Characteristic by category	Philippines (*N* = 444)	Malaysia (*N* = 463)	*p*-value^
**Sociodemographic**			
% female	73.4	72.7	0.845
Mean age (years)	55.6	59.7	< 0.001
% with post-secondary education	63.9	46.8	< 0.001
% married or cohabitating	72.7	73.1	0.895
% currently employed	44.8	20.5	< 0.001
Mean household size	4.5	3.8	0.012
Median monthly household income (local currency units)*	11,000 PHP	1500 MYR	
**Hypertension and health-seeking**			
Mean number of years since hypertension diagnosis	7.6	8.3	0.810
Mean systolic blood pressure (mmHg)	150.9	146.1	0.050
Mean diastolic blood pressure (mmHg)	93.1	88.2	< 0.001
% with hypertension controlled	27.2	28.9	0.532
% currently using antihypertensive medications	82.4	88.4	0.019
% of current antihypertensive medication users reporting good adherence ~	65.3	98.8	< 0.001
% who believe conventional medicine is effective to treat hypertension	88.2	81.8	0.042
% with good knowledge of hypertension	39.6	58.9	< 0.001
% with self-reported NCD comorbidities	28.8	53.7	< 0.001
% visiting a health provider at least 2 times per year for any reason	54.6	85.2	< 0.001
% that trust the health system	91.4	94.3	0.158
% that believe they can do something to maintain health	94.4	84.9	0.040
Mean self-reported life satisfaction score (1–10, least to most satisfied)	6.1	7.9	< 0.001
**TCAM**			
% currently using TCAM for hypertension	18.8	8.8	< 0.001
% using TCAM concurrently with antihypertensive medication^+^	82.4	71.6	< 0.001
% using TCAM only (and not antihypertensive medication) ^+^	17.6	28.4	0.219
% who were advised to take TCAM when diagnosed with hypertension	6.1	14.4	0.112
% who believe TCAM is effective to treat hypertension	54.9	21.0	< 0.001
Mean % of per capita total household expenditure spent on TCAM for any reason**	0.3	1.4	0.243
Mean % of per capita household health expenditure spent on TCAM for any reason**	7.0	12.1	0.446

^Wald test for a difference in proportions/means (weighted for sampling probability and adjusted for community-level clustering)

^~^N for this indicator is number aware hypertensive adults currently using antihypertensive medications

^+^N for this indicator is number aware hypertensive adults currently using TCAM for hypertension

^*^N is lower for this indicator due to refusals and 'don't know' responses

^**^N for this indicator is lower in Malaysia as expenditure data was collected from a sub-sample of enrolled households

More participants in Malaysia had good hypertension-related knowledge than in the Philippines (58.9% vs. 39.6%, *p* < 0.001), reported more co-morbidities (53.7% vs. 28.8%, *p* < 0.001), and visited a health provider at least twice per year (85.2% vs. 54.6%, *p* < 0.001). Trust in the health system was high in both countries (91.4% in the Philippines and 94.3% in Malaysia), as was the belief that one could do something to maintain health (94.4% vs. 84.9%). On the other hand, mean self-reported life satisfaction score was higher in Malaysia compared to the Philippines (7.9 vs. 6.1 out of 10, *p* < 0.001).

There were significantly more participants in the Philippines than Malaysia who reported using TCAM currently for hypertension (18.8% vs. 8.8%, *p* < 0.001), the majority of whom were using TCAM concurrently with antihypertensive medications (82.4% in the Philippines and 71.6% in Malaysia, *p* < 0.001). A much higher proportion in the Philippines believed that TCAM is effective in treating hypertension compared to Malaysia (54.9% vs. 21.0%, *p* < 0.001).

#### Types of TCAM used in Malaysia and the Philippines

Natural and biologically-based therapies were the most commonly mentioned category of TCAM used for hypertension in both countries, comprising 90.6% of mentions in the Philippines and 56.8% in Malaysia (Tables 2 and 3). However, the most frequently mentioned TCAM modalities used in Malaysia were massage (21.6%) and cupping (16.2%), both manipulative and body-based treatments; while in the Philippines, Annona muricata [guyabano (soursop)] (20.3%), Cymbopogon sp. [lemongrass] (19.5%), Rauvolfia serpentina [serpentina] (12.3%) and Blumea balsamifera [sambong] (11.3%), all natural and biologically-based treatments, were most frequently mentioned. Participants did not mention modalities related to mind–body therapies in either country; and while some specific mentions, such as acupuncture, may fall within the cross-cutting domain related to whole medical systems, participants did not mention any specific system.

**Table 2:** Distribution of mentions (*n* = 212) of TCAM modalities used for hypertension in the Philippines

Type of TCAM (by domain)	% of mentions
**Natural and biologically-based products**	**90.6**
Annona muricata (guyabano/soursop) leaves	20.3
Cymbopogon sp. (lemongrass) leaves, tea, roots	19.8
Rauvolfia serpentina (serpentina) leaves	12.3
Blumea balsamifera (sambong) leaves	11.3
Pandanus amaryllifolius (pandan) leaves, tea	3.8
Cymbopogon citratus (tanglad) leaves	3.3
Lagerstroemia speciosa (banaba) leaves	2.8
Allium sativum (garlic) oil, tea, with honey, capsules	2.8
Coleus amboinicus (oregano) leaves	2.8
Eleusine indica (paragis) leaves, grass, tea	2.8
Vitex negundo (lagundi) leaves	2.4
Moringa oleifera (malunggay) leaves	2.4
Musa sp. (banana) leaves	1.9
Ehretia microphylla (tsaang gubat), tea or juice	1.9
**Manipulative and body-based systems**	**7.5**
Massage	6.1
Hilot (traditional Filipino massage)	0.5
Physical Therapy	0.5
Reflexology	0.5
**Energy therapies**	**0.5**
Acupuncture	0.5
**Other non-medical, non-herbal**	1.4

**Table 3:** Distribution of mentions (*n* = 37) of TCAM modalities used for hypertension in Malaysia

TCAM type (by domain)	% of mentions
**Manipulative and body-based systems**	**40.5**
Massage	21.6
Cupping	16.2
Ceragam™ thermal massage	2.7
**Energy therapies**	**2.7**
Acupuncture	2.7
**Natural and biologically-based**	**59.5**
Non-specific root (akar kayu) drinks	8.1
Bitter melon/gourd (peria katak) juice, capsules	5.4
Al Sharia™ herbal medicine	2.7
Andrographis paniculate (hempedu bumi) leaves	2.7
Annona muricata (soursop) juice	2.7
Cynometra cauliflora (nam nam) fruit	2.7
Eskayvie phytax™ drink of various fruit extracts	2.7
Fig vinegar	2.7
Gardenia augusta (kacapiring/jasmine) leaves	2.7
Genus stichopus (Gamat/sea cucumber)	2.7
Herbal juice	2.7
Herbalife™ product with protein powder, fruit extracts, herbs	2.7
Honey	2.7
Javanese sour herbs	2.7
Olive oil	2.7
Phaleria macrocarpa (Mahkota dewa) fruit	2.7
Physalis angulata (letup-letup) leaves	2.7
Pil mujarab (non-specific herbal tablet)	2.7
Strobilanthen crispa (pecah kaca) leaves	2.7

#### Determinants of TCAM use

In both countries, participants who believed that TCAM is effective as hypertension treatment were more likely to use it (Table 4). Conversely, those who believed in conventional medicine were less likely to use TCAM; however, strong evidence for this association was observed only in the Philippines (aOR: 0.30, 95%CI: 0.15–0.62), but not in Malaysia (aOR: 0.55, 95%CI: 0.23–1.36). In Malaysia only, there was strong evidence that being diagnosed with hypertension for at least 5 years (aOR: 0.44, 95%CI: 0.20–1.00) and increasing household wealth (aOR: 0.70, 95%CI: 0.53–0.92) decreased the likelihood of TCAM use, while having any post-secondary education increased this likelihood (aOR: 2.82, 95%CI: 1.20–6.62). The adjusted point estimates in both countries suggest that females are more likely to use TCAM, but without strong statistical evidence. Strong evidence that country is an effect modifier was noted for the adjusted associations between TCAM use and time since diagnosis (*p* = 0.023) and education (*p* = 0.037). Sex, age, marital status, employment status, trust in the health system, self-efficacy, urban–rural location, good knowledge of hypertension or self-reported co-morbidities were not significantly associated with TCAM use for hypertension.

**Table 4:** Determinants of TCAM use, by country (odds ratios with [95% confidence intervals])

Determinant		Philippines (*N* = 444)		Malaysia (*N* = 463)	
		**Crude**	**Adjusted**	**Crude**	**Adjusted**
Believes TCAM is effective	No	1	1	1	1
	Yes	2.54***	3.19***	5.97***	6.79***
		[1.47,4.37]	[1.78,5.72]	[2.93,12.16]	[3.12,14.77]
Believes conventional medicine is effective	No	1	1	1	1
	Yes	0.38**	0.30**	0.50	0.55
		[0.20,0.72]	[0.15,0.62]	[0.23,1.07]	[0.23,1.36]
Good knowledge of hypertension	No	1	1	1	1
	Yes	1.02	1.02	0.67	0.93
		[0.63,1.66]	[0.60,1.75]	[0.34,1.34]	[0.43,2.01]
Diagnosed with hypertension for at least 5 years	No	1	1	1	1
	Yes	1.15	1.36	0.43*	0.44*
		[0.71,1.87]	[0.81,2.30]	[0.21,0.87]	[0.20,1.00]
Self-reported history of NCD comorbidity	No	1	1	1	1
	Yes	1.21	1.25	0.80	1.07
		[0.70,2.08]	[0.70,2.26]	[0.40,1.58]	[0.49,2.34]
Visits health provider at least 2 times per year for any reason	No	1	1	1	1
	Yes	1.66*	1.57	0.71	1.01
		[1.01,2.72]	[0.92,2.68]	[0.29,1.72]	[0.33,3.06]
Female	No	1	1	1	1
	Yes	1.38	1.50	1.22	2.31
		[0.79,2.42]	[0.82,2.72]	[0.53,2.78]	[0.80,6.66]
Age 50 + years	No	1	1	1	1
	Yes	1.18	1.06	0.65	0.85
		[0.67,2.10]	[0.56,1.99]	[0.24,1.79]	[0.26,2.84]
Any post-secondary education	No	1	1	1	1
	Yes	0.85	0.96	2.52*	2.82*
		[0.52,1.38]	[0.56,1.66]	[1.21,5.25]	[1.20,6.62]
Married/cohabitating	No	1	1	1	1
	Yes	1.04	1.26	0.90	0.93
		[0.61,1.77]	[0.71,2.25]	[0.43,1.90]	[0.37,2.38]
Currently employed	No	1	1	1	1
	Yes	0.63	0.68	1.69	1.38
		[0.39,1.02]	[0.40,1.17]	[0.77,3.73]	[0.51,3.77]
Has confidence in health system	No	1	1	1	1
	Yes	0.76	0.94	2.75	2.21
		[0.38,1.52]	[0.43,2.04]	[0.36,21.01]	[0.27,18.33]
Believes one can do something to maintain health	No	1	1	1	1
	Yes	0.72	0.89	2.12	2.94
		[0.26,2.03]	[0.29,2.69]	[0.62,7.23]	[0.74,11.63]
Rural location	No	1	1	1	1
	Yes	1.06	1.13	1.81	1.72
		[0.66,1.71]	[0.62,2.05]	[0.80,4.12]	[0.77,3.83]
Household wealth score		0.98	0.95	0.76*	0.70*
		[0.86,1.12]	[0.81,1.12]	[0.61,0.95]	[0.53,0.92]
Constant			0.15*		0.00***
			[0.02,0.99]		[0.00,0.10]

Evidence from Wald tests for the equality of adjusted coefficients by country in the combined dataset was observed for ‘Diagnosed with hypertension for at least 5 years’ (*p* = 0.023), and ‘Any post-secondary education’ (*p* = 0.037)

^*^p < 0.05

^**^p < 0.01

^***^p < 0.001

#### Effect of TCAM use on hypertension management and wellbeing

Our analysis did not find strong evidence for any of the associations between TCAM use and the use of blood pressure-lowering medications, self-reported adherence to that medication, blood pressure level and control, or wellbeing in either country, or for any significant differences across the two countries (Table 5).

**Table 5:** Associations of TCAM use with hypertension outcomes and wellbeing (odds ratios or regression coefficients with [95% confidence intervals])

Binary outcomes	Philippines		Malaysia	
	**Crude odds ratio**	**Adjusted odds ratio**	**Crude odds ratio**	**Adjusted odds ratio**
Current antihypertensive medication use	1.05 [0.58, 1.91]	1.07 [0.56, 2.06]	0.41 [0.18, 0.91]	1.00 [0.32, 3.12]
Antihypertensive medication adherence^+^	0.75 [0.42, 1.36]	0.67 [0.34, 1.31]	0.28 [0.03, 2.59]	0.30 [0.02, 3.89]
Hypertension control	0.68 [0.37, 1.24]	0.75 [0.40, 1.42]	0.55 [0.23, 1.32]	0.69 [0.27, 1.77]
**Continuous outcomes**	**Crude coefficient**	**Adjusted coefficient**	**Crude coefficient**	**Adjusted coefficient**
Systolic blood pressure (mmHg)	1.97 [-3.80, 7.74]	0.19 [-5.50, 5.88]	8.97 [0.06, 17.88]	3.35 [-5.40, 12.09]
Diastolic blood pressure (mmHg)	0.91 [-2.50, 4.32]	0.23 [-3.07, 3.52]	4.96 [-0.38, 10.30]	1.29 [-3.61, 6.18]
Wellbeing (1–10, most to least satisfied)	0.23 [-0.41, 0.88]	0.37 [-0.28, 1.03]	-0.05 [-0.57, 0.48]	-0.16 [-0.69, 0.37]

^+^Model for adherence is restricted to participants who reported current use of antihypertensive medication. No evidence from Wald tests for the equality of adjusted coefficients by country in the combined dataset was noted. All models are adjusted for belief in the effectiveness of TCAM and conventional medicine for hypertension, knowledge of hypertension, time since diagnosed with hypertension, history of NCD comorbidity, frequency of health provider visits, sex, age, education, marital/co-habitation status, employment, confidence in health system, belief that one can do something to maintain health, urban–rural location, household wealth. Models assessing the association between hypertension control, systolic and diastolic blood pressure and wellbeing and TCAM use are further adjusted for current use of antihypertensive medication. Appendix 5 in the Supplemental information provides the full results for each model

^*^p < 0.05

^**^p < 0.01

^***^p < 0.001

## Discussion

In this study of hypertensive adults living in low-income communities in two middle-income Southeast Asian countries, 9% in Malaysia and 19% in the Philippines reported that they were currently using some form of TCAM for hypertension, most of whom were using it to complement their treatment with conventional medications (Table 1).

Making comparisons with other literature is challenging due to differences in how TCAM use is measured, and our focus on hypertension and on the poorest communities [2, 36]. What we observed is considerably lower than what has been seen in the general populations of both Malaysia (56%) and the Philippines (43%), [1, 15] however, these estimates capture *any use over the past 6 or 12 months for any reason*, as opposed to our estimates of *current use (i.e. in the past 2 weeks) for hypertension*. Similarly, our figures are lower than what has been observed among hypertensive primary care patients both in Thailand and Cambodia, 33% of whom used *herbal medicine over the past 12 months*, as opposed to *use in the past 2 weeks of any TCAM modality* as in our metric; [27, 42] and also in Malaysia, 27% of whom reported using any TCAM [30]. Our observations are, however, broadly consistent with national estimates among people diagnosed with hypertension from the WHO STEPS surveys in the region, which found that 15% in Cambodia, 13% in Myanmar, but also 3% in Laos *consulted TCAM providers for any reason during the previous 12 months* (as opposed to *used any TCAM modality for hypertension* specifically) [3].

Although our samples include only low-income households and women are over-represented – both of which are known predictors of TCAM use and would, therefore, predict higher rates of use [10] – our lower estimates are not entirely unexpected. Other RESPOND study findings have clearly demonstrated that hypertension is widely viewed as a minor health condition that requires treatment with medications only when experiencing symptoms [43]. This general attitude toward treatment is likely to extend to the use of TCAM for hypertension, predisposing a lower rate of current TCAM use given the largely asymptomatic nature of hypertension. Also, qualitative findings indicate that a major reason patients use TCAM is to manage the side effects of conventional antihypertensive medications when they arise, [44, 45] and therefore, may only be used intermittently.

What is perhaps more telling is that the prevalence of TCAM use for hypertension in the Philippines is double what was observed in Malaysia. Our analysis of the determinants of TCAM use showed that belief in the effectiveness of TCAM for hypertension treatment increased the likelihood of use in both countries (although, we cannot rule out the possible effect of reverse causality in this observation as a cross-sectional study). Nevertheless, the much higher proportion of Filipinos who held this belief compared to in Malaysia (55% vs. 21%) is a clear driver of this difference. There may be other important predisposing factors underlying this difference that we did not examine in our study, such as the level of satisfaction with conventional medicine, and the influence of friends and family [2, 46].

The mix of commonly used TCAM modalities is also likely to underlie this cross-country difference in the prevalence of TCAM use, which is, in turn, a product of individual preferences, and the modalities available and affordable to them. Natural and biologically-based therapies were the most commonly mentioned form in both countries, comprising 91% of mentions in the Philippines, but only 57% in Malaysia; and most of these mentions referred to herbal remedies that may be affordably purchased or grown in gardens for self-administration. This predominance of herbal treatments for hypertension has also been noted in studies from Cambodia, [42] Laos, [28] Myanmar, [26] and Thailand [25, 27]. While in Malaysia, more costly provider-based treatments, namely massage and cupping, were also often used (Tables 2 and 3). This preference for more costly provider-based treatments is also reflected in the observed levels of TCAM expenditures (Table 1), where Malaysian households tended to allocate more of their total household and health budgets on TCAM, despite their lower use. Yet overall, spending on TCAM by low-income households in both countries was relatively low, meaning that it not likely to be a significant driver of health or social inequality.

Finally, the difference in TCAM use for hypertension may also reflect lower levels of available, affordable and acceptable conventional primary care services and medications for hypertension in the Philippines, when compared to Malaysia. For example, despite high levels of confidence in the overall health system in both countries, only about half in the Philippines (55%) reported visiting a health provider at least 2 times per year for any reason compared to 85% in Malaysia, reflecting a crucial difference in realised access to or actual use of conventional care. Realised access accounts for both the care that is on offer and whether the individual is willing and able to use it [47]. A number of known supply-side drivers of TCAM use related to dissatisfaction with conventional healthcare, such as long distance to facilities, unavailability of medicines, negative attitude of healthcare providers and long waiting times, [2, 40] are all more prevalent in the Philippines [18, 20].

Our finding that TCAM is mainly being used concurrently with, rather than as an alternative to conventional medication, which has also been reported in other Southeast Asian studies, [29, 30] is potentially encouraging. This is also consistent with our observation that TCAM use was not associated with decreased (or increased) use of conventional antihypertensive medication (Table 5). As noted above, qualitative findings from both countries indicate that many use TCAM concurrently to relieve the perceived side effects of their conventional medications [44, 45]. However, among the forms that were used in our study population, namely a wide range of herbal treatments, massage, cupping and acupuncture, evidence on their effectiveness at lowering blood pressure as a complementary or alternative treatment to antihypertensive medications is either of low quality, conflicting or suggestive of no benefit [48–52].

Of particular concern is the lack of safety data on such treatments when used either alone on as an adjuvant to conventional hypertension medications. Thus, addressing this knowledge gap provides a clear justification for the national TCAM policies, regulations and institutions enacted in many countries, including Malaysia and the Philippines, and a focus for the work on establishing the efficacy, adverse effects and drug interactions that they must do. In the interim, the absence of such safety data must guide regulation of the marketing of these treatments, and should be highlighted in clinical and public health messaging to empower individuals to make more evidence-informed decisions about TCAM use for the management of hypertension.

While there may be little evidence on either the benefits or harms of TCAM for hypertension when used as an alternative or complementary to conventional treatments, there is strong evidence that TCAM use could reduce adherence to conventional blood pressure-lowering medication and, ultimately, the likelihood of achieving hypertension control, [53] which is also suggested by our study findings (Table 5). Qualitative findings from Malaysia may help to explain this negative association, which reveal a widely-held belief among TCAM practitioners that hypertension was curable and that traditional remedies for hypertension should not be taken concurrently with conventional medications [45]. Our inability to detect statistically significant associations between TCAM use and hypertension medication adherence and control is likely due to a lack of power. But in the case of adherence which is measured using self-reports, we also acknowledge that our estimates are affected by measurement error, as lay understandings of adherence and the chronic nature of hypertension are known to substantially differ from clinical ones [43, 54]. Hence, our levels of adherence are likely to be overestimated and the association between TCAM use and adherence is likely to be underestimated. Our finding that TCAM use was not associated with self-rated wellbeing is also consistent with evidence from the literature [55]. Therefore, clinical and public health messaging to hypertensive populations should aim to increase knowledge and understanding of the importance of long-term medication adherence for hypertension, especially in light of its typically asymptomatic – yet chronic – nature, along with the effectiveness of existing medications, which itself was a strong determinant of TCAM use.

One final consideration in interpreting our findings concerns their generalisability to other low-income communities in Malaysia and the Philippines, and to other LMICs. Although there were some notable differences in the characteristics of the participants included and excluded in this analysis (Appendix 3 in the Supplemental information), the small numbers of those excluded are unlikely to affect our estimates. Our results on the determinants of TCAM use in Malaysia align with those from a study of the rural Malaysian population, which also found belief in the effectiveness of TCAM, higher education levels and lower household wealth to be strong predictors [12] While this finding on education is inconsistent with other studies, particularly from LMICs, [2, 10] a similar positive association between TCAM use and education has been observed in various high-income settings, [56, 57] likely reflecting Malaysia’s current socio-economic status and trajectory. As previously noted, women were over-represented in our sample, likely reflecting their higher availability and/or willingness to participate; but this does not appear to have produced any inexplicable findings. Others have also suggested that TCAM use is likely to be underreported due to social desirability bias; [45, 58, 59] yet we have taken steps to minimise this by interviewing respondents in their homes (rather than in clinical settings) and by deploying non-medical professionals trained in non-judgemental interviewing techniques. On the other hand, we accept that participants may have still considered the study as ‘clinical’ because it involved blood pressure measurement at enrolment. The median household income, level of hypertension control, education and employment observed in our country samples are closely aligned with national data, [22, 60, 61] which suggests that we have, indeed, sampled a suitable cross-section of hypertensive adults in low-income communities.

## Conclusion

TCAM is used in the management of hypertension by a relatively small, but not insignificant, proportion of affected individuals living in low-income communities in Malaysia and the Philippines. Yet, evidence to establish the efficacy and safety of commonly used TCAM modalities, and on their potential interactions with conventional medications, is needed to ensure they are used for optimal benefit. In the interim, national health systems must continue to work to improve access to primary care services, and health care providers seeking to provide effective patient-centred care for hypertension must recognise that their patients may be receiving advice and treatment from a variety of sources that may influence their knowledge, understanding and, ultimately, the management of their condition.

## Supplementary information

**Additional file 1: Appendix 1.** Variable definitions. **Appendix 2.** Derivation of sampling probability weights. **Appendix 3.** Characteristics of participants included and excluded from the RESPOND study TCAM analysis. **Appendix 4.** STROBE checklist of items that should be included in reports of cross-sectional studies and sample derivation flow diagram. **Appendix 5.** Full model results for association of TCAM use with hypertension management outcomes and wellbeing.

## Data Availability

The dataset analysed for the current study are not publicly available due to concerns about the privacy and confidentiality of participant data, but are available from the corresponding author on reasonable request, subject to approval of the RESPOND study co-investigators.

## Abbreviations

aOR: Adjusted odds ratio
ASEAN: Association of Southeast Asian Nations
cOR: Crude odds ratio
CVD: Cardiovascular disease
DBP: Diastolic blood pressure
LMIC: Low- and middle-income country
MYR: Malaysian Ringgit
NCD: Non-communicable disease
PHP: Philippine Peso
RESPOND: Responsive and Equitable Health Systems – Partnership on NCDs
SBP: Systolic blood pressure
TCAM: Traditional and complementary/alternative medicine
WHO: World Health Organization
95%CI: 95% Confidence interval
