# Supplementary material for: Prevalence, determinants and outcomes of traditional, complementary and alternative medicine use for hypertension among low-income households in Malaysia and the Philippines
Source: BMC Complement Med Ther. 2022 Sep 30;22:252. doi: 10.1186/s12906-022-03730-x (PMC9526286; doi:10.1186/s12906-022-03730-x)
Supplement: Supplementary file 1 — Additional file 1: Appendix 1. Variable definitions. Appendix 2. Derivation of sampling probability weights. Appendix 3. Characteristics of participants included and excluded from the RESPOND study TCAM analysis. Appendix 4. STROBE checklist of items that should be included in reports of cross-sectional studies and sample derivation flow diagram. Appendix 5. Full model results for association of TCAM use with hypertension management outcomes and wellbeing. [file 12906_2022_3730_MOESM1_ESM.zip › Supplementary information - Appendices 1-4.docx]

# SUPPLEMENTARY INFORMATION

## Appendix 1: Variable definitions

| **TCAM use outcome** | |
| --- | --- |
| TCAM use | Self-reported to be currently using any form of traditional, complementary or alternative treatment or service to manage hypertension among all diagnosed hypertensives (no vs. yes) |
| **Hypertension management and health outcomes** | |
| Treatment | Self-reported to be currently using a specific antihypertensive medication among all diagnosed hypertensives (no vs. yes) |
| Control | Those who had an average (across 3 measurements) systolic and diastolic blood pressure of less than 140/90 mmHg among all diagnosed hypertensives (no vs. yes) |
| Adherence | Self-reported to be currently adhering to their prescribed antihypertensive medication among those currently using such medication (no vs. yes) |
| Systolic blood pressure | Average systolic blood pressure across 3 measurements (mmHg) |
| Diastolic blood pressure | Average diastolic blood pressure across 3 measurements (mmHg) |
| Wellbeing | Self-reported overall current satisfaction with life (scale ranging from 1=least satisfied to 10=most satisfied) |
| **Individual and household determinants and covariates** | |
| Believes TCAM is effective to treat hypertension | no vs. yes |
| Believes conventional medicine is effective to treat hypertension | no vs. yes |
| Good knowledge of hypertension | Correctly answered at least 4 out of 5 questions about the consequences (can cause stroke or cancer), symptoms (may be asymptomatic) and management of hypertension (medication needed only when blood pressure is high or feel unwell) (no vs. yes) |
| Diagnosed with hypertension for at least 5 years | no vs. yes |
| Self-reported history of NCD comorbidity | Self-reported history of any of the following non-communicable diseases: diabetes mellitus, stroke, heart attack, heart failure, cancer, chronic obstructive pulmonary disease, asthma, valvular heart disease (no vs. yes) |
| Visits regular health provider at least 2 times per year for any reason | no vs. yes |
| Female | Male vs. female sex |
| Age | Current age in years is below 50 vs. 50 years or higher |
| Education | Completed only primary, secondary or no education vs. completed any post-secondary education/trade school |
| Married/cohabitating | Never married/widowed/separated/divorced vs. currently married/common law/living with partner |
| Currently employed | no vs. yes |
| Has confidence in health system | no vs. yes |
| Believes one can do something to maintain health | no vs. yes |
| Location | Rural vs. urban location |
| Household wealth | Normalised country-specific asset-based wealth score derived using principal components analysis |
| Country | Philippines vs. Malaysia |
| **Other variables** | |
| Household income | Self-reported from all sources in local currency (Malaysian Ringgit, MYR; Philippine Peso, PHP) |
| Household size | no vs. yes |
| Per capita total household expenditure spent on TCAM for any reason | Annualised monthly household expenditure on TCAM for any reason over annualised total monthly household expenditure (%) |
| % of per capita household health expenditure spent on TCAM for any reason | Annualised monthly household expenditure on TCAM for any reason over annualised total monthly household health expenditure (%) |
| Using TCAM concurrently with antihypertensive medication | Self-reported to be currently using any form of traditional of complementary medicine or treatment to manage hypertension *and* antihypertensive medication among all diagnosed hypertensives (no vs. yes) |
| Using TCAM only (and not antihypertensive medication) | Self-reported to be currently using any form of traditional of complementary medicine or treatment to manage hypertension *and not any* antihypertensive medication among all diagnosed hypertensives (no vs. yes) |
| Advised to take TCAM when diagnosed with hypertension | Self-reported to have be advised to take any form of traditional of complementary medicine or treatment to manage hypertension *at the time of diagnosis* among all diagnosed hypertensives (no vs. yes) |

## Appendix 2: Derivation of sampling probability weights

Sampling weights are needed to analyse the household survey data to avoid biased estimates due to the multi-stage sampling approach that selected communities in each rural and urban strata with probability proportional to their varying population size, an equal number of households within each community, and one household member to participate among all those eligible within the household. Individual-level weights are calculated by taking the inverse product of the:

- Unconditional probability of selecting the community (mukim/barangay) within the state/province (e.g. mukim population/state population at the time of data collection)
- Conditional probability of selecting household within the community (e.g. number of households screened in mukim/total number of households in mukim)
- Conditional probability of selecting participant from all eligible residents within household (i.e. 1/number of eligible adults screened within the household)

## Appendix 3: Characteristics of participants included and excluded from the RESPOND study TCAM analysis

|  | **Philippines** | | | **Malaysia** | | |
| --- | --- | --- | --- | --- | --- | --- |
| **Characteristic** | **Excluded** | **Included** | **p-value^** | **Excluded** | **Included** | **p-value^** |
| Number of aware hypertensive adults (N) | 7 | 444 |  | 32 | 463 |  |
| % female | 63·5 | 73·4 | 0·116 | 88·5 | 72·7 | 0·010 |
| Mean age (years) | 57·6 | 55·6 | 0·038 | 54·8 | 59·7 | 0·001 |
| % with post-secondary education | 18·4 | 63·9 | 0·000 | 50·6 | 46·8 | 0·415 |
| % married or cohabitating | 72·4 | 72·7 | 0·974 | 64·3 | 73·1 | 0·134 |
| % currently employed | 36·8 | 44·8 | 0·251 | 34·4 | 20·5 | 0·307 |
| Mean number of years since hypertension diagnosis | 8·2 | 7·6 | 0·812 | 8·1 | 8·3 | 0·862 |
| % with self-reported NCD comorbidities | 23·2 | 28·8 | 0·074 | 34·8 | 53·7 | 0·000 |

*^Wald test for a difference in proportions/means (weighted for sampling probability and adjusted for community-level clustering)*

## Appendix 4: STROBE checklist of items that should be included in reports of *cross-sectional studies* and sample derivation flow diagram

|  | Item No | Recommendation | Page No |
| --- | --- | --- | --- |
| Title and abstract | 1 | (*a*) Indicate the study’s design with a commonly used term in the title or the abstract | p.1-2 |
|  |  | (*b*) Provide in the abstract an informative and balanced summary of what was done and what was found | p. 1-2 |
| Introduction | | | |
| Background/ rationale | 2 | Explain the scientific background and rationale for the investigation being reported | p. 2-4 |
| Objectives | 3 | State specific objectives, including any prespecified hypotheses | p. 4-5 |
| Methods | | | |
| Study design | 4 | Present key elements of study design early in the paper | p. 5-6 |
| Setting | 5 | Describe the setting, locations, and relevant dates, including periods of recruitment, exposure, follow-up, and data collection | p. 5-7 |
| Participants | 6 | (*a*) Give the eligibility criteria, and the sources and methods of selection of participants | p. 6-7 |
| Variables | 7 | Clearly define all outcomes, exposures, predictors, potential confounders, and effect modifiers. Give diagnostic criteria, if applicable | p. 8-9 Appendix 1 |
| Data sources/ measurement | 8* | For each variable of interest, give sources of data and details of methods of assessment (measurement). Describe comparability of assessment methods if there is more than one group | p. 7-9 Appendix 1 |
| Bias | 9 | Describe any efforts to address potential sources of bias | p. 6,9-10,16-17 |
| Study size | 10 | Explain how the study size was arrived at | p. 7 |
| Quantitative variables | 11 | Explain how quantitative variables were handled in the analyses. If applicable, describe which groupings were chosen and why | p. 7-9 Appendix 1 |
| Statistical methods | 12 | (*a*) Describe all statistical methods, including those used to control for confounding | p. 9-10 |
|  |  | (*b*) Describe any methods used to examine subgroups and interactions | p. 9-10 |
|  |  | (*c*) Explain how missing data were addressed | p. 10-11 |
|  |  | (*d*) If applicable, describe analytical methods taking account of sampling strategy | p. 9-10, Appendix 2 |
|  |  | (*e*) Describe any sensitivity analyses | p. 10, Appendix 3 |
| Results | | | |
| Participants | 13* | (a) Report numbers of individuals at each stage of study—eg numbers potentially eligible, examined for eligibility, confirmed eligible, included in the study, completing follow-up, and analysed | p. 10-11 |
|  |  | (b) Give reasons for non-participation at each stage | p. 10-11 |
|  |  | (c) Consider use of a flow diagram | Appendix 4 |
| Descriptive data | 14* | (a) Give characteristics of study participants (eg demographic, clinical, social) and information on exposures and potential confounders | p. 11, Table 1 |
|  |  | (b) Indicate number of participants with missing data for each variable of interest | p. 10-11 |
| Outcome data | 15* | Report numbers of outcome events or summary measures | Tables 2 to 5 |
| Main results | 16 | (*a*) Give unadjusted estimates and, if applicable, confounder-adjusted estimates and their precision (eg, 95% confidence interval). Make clear which confounders were adjusted for and why they were included | Tables 4 and 5, Appendix 5 |
|  |  | (*b*) Report category boundaries when continuous variables were categorized | Table 4, Appendix 5 |
|  |  | (*c*) If relevant, consider translating estimates of relative risk into absolute risk for a meaningful time period | N/A |
| Other analyses | 17 | Report other analyses done—eg analyses of subgroups and interactions, and sensitivity analyses | Appendix 3 |
| Discussion | | | |
| Key results | 18 | Summarise key results with reference to study objectives | p. 13 |
| Limitations | 19 | Discuss limitations of the study, taking into account sources of potential bias or imprecision. Discuss both direction and magnitude of any potential bias | p. 13-17 |
| Interpretation | 20 | Give a cautious overall interpretation of results considering objectives, limitations, multiplicity of analyses, results from similar studies, and other relevant evidence | p. 13-17 |
| Generalisability | 21 | Discuss the generalisability (external validity) of the study results | p. 16-17 |
| Other information | | | |
| Funding | 22 | Give the source of funding and the role of the funders for the present study and, if applicable, for the original study on which the present article is based | p. 19 |

1191 screened to have hypertension in RESPOND project

(Philippines = 606; Malaysia = 585)

39 Excluded due to missing data

(Philippines = 7; Malaysia = 32)

907 included in analysis

(Philippines = 444; Malaysia = 463)

946 diagnosed to have hypertension by health professional

(Philippines = 451; Malaysia = 495)

## Appendix 5: Full model results for association of TCAM use with hypertension management outcomes and wellbeing

[See provided file]
